# Dry eye among computer operators at a tertiary care centre in India

**DOI:** 10.6026/97320630018912

**Published:** 2022-10-31

**Authors:** MC Chaitra, J Akhil, Kunchapu Meghana, SJ Lavanya, YS Navya, Bhimagani Lathasri

**Affiliations:** 1 Sri Devaraj Urs Medical College, Constituent of Sri Devaraj Urs Academy of Higher Education and Research, Kolar, Karnataka, India

**Keywords:** Dry Eye, Schirmer Test, Tear Break-up Time, Ocular Surface Disease Index, Computer operators

## Abstract

There is global digitalization. With the increased use of computers in the workplace, refraction and dry eye related health concerns are developing. This study is conducted to evaluate the effects of long-term computer use on tear production and evaporation. Thisis a cross-sectional study among 97 computer operators in a Tertiary care centre. Dry eye disease was assessed subjectively with the Ocular Surface Disease Index (OSDI) questionnaire and objectively by performing Schirmer's test and Tear Break-up Time (TBUT). Majority of participants (59.2%) were male's age group between 36- 40 years. The prevalence of dry eye among the computer Operators was found to be 51%. Mean TBUT and mean Schirmer’s Test value for those with dry eye was 12.65s and 10.3mm respectively. This study showed that a positive relation between dry eye and computer usage. Computer users are predisposed to developing dry eye diseases. It is therefore necessary for them to go for regular ophthalmic examination to prevent dry eye symptoms and disease.

## Background:

Dry eye is a common disease estimated to have affected 25-30 million people all over the world. [1, 2] Males and females have been associated with the risk of developing the dry eye disease. However, females are at a greater risk due to deficient tear secretion from estrogen deficiency with increasing age. [3, 4] Dry eye can cause damage to the intra-palpebral ocular surface and is associated with a variety of symptoms such as eye strain, redness of eyes, dry eyes, tiredness, itching, blurring, or double vision, reflecting ocular discomfort. [5] Additionally, patients with dry eye are prone to potentially blinding infections, like bacterial keratitis & increased risk of complications following laser refractive surgery. Extended visual tasking during computer use, television watching and prolonged reading could provoke the symptoms of dry eye disease. [6] This has been attributed to the decrease in blink rate while using these devices and also linked to decreased mucin concentration. [7] Therefore, it is of interest to evaluate the effects of long-term computer use on tear production and evaporation among computer operators/clerks, who are constantly exposed to computer screen as per their job profile.

## Methodology:

### Study design:

A Cross sectional study.

### Study duration:

January 2022 to April 2022.

### Study population:

Computer operators working at Sri Devaraj Urs MedicalCollege, RL Jalappa Hospital & Research Centre, constituents of Sri DevarajUrs Academy of Higher Education & Research,Tamaka, Kolar.

### Sample size:

97 Sample size was estimated based on prevalence of dry eyes 74% as reported in a study. Considering an absolute error of 10% with 95% confidential interval of sample size required for study is 97 people working with computers.

Inclusion criteria:

[1] Both male and female subjects

[2] Between the age of 20 to 40 years of age

[3] Computer users using computer for more than 2 hours per day at least since 1 year.

[4] Willing to give prior consent for evaluation.

Exclusion Criteria:

[1] Subjects with history of allergic conjunctivitis, gross lid abnormalities, life threatening systemic disease, acute ocular infections, extra and intraocular surgery within last 6 months.

[2] Subjects taking systemic medications or ocular topical medications known to cause dry eyes like antihistaminic, anticholinergic, antiglaucoma, topical steroids, etc

[3] Contact lens users

After obtaining Ethical clearance from the Institutional Ethics Committee, informed consent from the participants was taken. Demographic details were noted. Subjects who fulfilled the inclusion criteria were administered the Ocular Surface Disease Index (OSDI) questionnaire. The OSDI questionnaire had a total of 12 questions on dry eyes and was subdivided into 3 sections. The first section was regarding the symptoms of dry eye, second was on the effects of dry eye and the third section was on aggravating symptoms. The OSDI score was evaluated as (A+B+C) x 25 / N (where N was the number of questions what were answered). Higher score representing the greater disability. [8] Tear quality and quantity were then evaluated using tear breakup time test and Schirmer I test respectively.

In the Schirmer I test, the strip was placed in the lateral one-third of the lower eyelid. After 5 minutes, the strip was removed and the level of wetting (in millimetres) was noted. [9] For Tear break up time (TBUT), Fluorescein was applied to the lower part of the bulbar conjunctiva and the subject was told to blink to spread the fluorescein. The tear film was assessed under cobalt blue light and the interval from the last blink to the appearance of the first dry eye spot on the cornea noted. Data obtained was analyzed using SPSS and Descriptive analysis such as percentages, and tables are used to summarize the data. Using Craig et al.[9, 10] criteria, subjects with an OSDI score of ≥35 and who had < 10mm to Schirmer's I test or < 10secs in Tear break-up Time (TBUT) test were considered to have dry eye.

## Results:

A total number of 97 computer operators were examined in Sri Devaraj Urs Academy of Higher education and Research, out of which 59.2% were males and 40.8% were females.37.1% participants belonged to age group of 36-40 years, followed by 20.6% - 31-35years, 19.6 % belonged to 26-30 years, 9.3% between 41-45 years, 7.2% belonged to <25 years and 6.2 % were > 45 years (Table 1 - see PDF). Overall Prevalence of Dry eye was found to be 51%.In our study the mean ocular surface disease index score was 39. When the OSDI Score cut off value is set at ≥35 the prevalence of dry eye is 41%. (Figure 1 - see PDF) Mean Schirmer-1 test values in right eye was found to be 10mm and in left eye 10.6mm. (Figure 2 and Table 2 - see PDF) The mean tear film break up time in right eye was found to be 12.3seconds and in left eye 13 seconds. (Figure 3 and Table 3 - see PDF)

## Discussion:

Studies conducted in various location of India found prevalence rates in high exposure occupational groups such as computer users, drivers etc. in a study by Choudhary et al. = 12.3 %, Sahai et al. = 20.7%, Shah et al. = 58.6% and Ranjan et al. 61.54% was the prevalence of dry eye. [11-12] Hagan et al. conducted a study on 112 non contact lens using computer operators and found that 68% men and 73% women had symptoms of dry eye. [13] From the above studies it can be said that the prevalence of dry eye among high risk groups ranges from 20 % to 73%. In our study prevalence among the computer Operators was found to be 51%. 31 to 40 years of age group was predominantly high suggesting that the prevalence of dry eye increases as the years of computer exposure increases. Tsubota [14] had shown that the mean blink rate significantly drops down as level of concentration or attention increases. Mean blink rate is 22 per min in relaxed state to 10 per min when reading a book and 7 per min on the computers. Since the main route of tear elimination is through evaporation, longer periods of eye opening and the higher gaze angle when viewing a computer screen results in faster tear loss which further worsens the dry eye. [15] Ergonomic position -one arm distance or 40 inches away with a downward gaze of 14° or more appears to help relieve the symptoms of computer related dry eye. Avoid screen reflections, glare from window, or overhead lights. Avoid turning the air conditioning too high or direct draughts to the face. [16] We have studied subjective symptoms using a pre validated questionnaire like OSDI questionnaire and objective tests of tear film quality, and quantity to make a diagnosis of dry eye syndrome. We have found a good association between the subjective symptoms questionnaire and objective test for tear film. It seems that the prevalence of dry eye is increasing in the era of the Internet. Thus, as ophthalmologists will probably encounter an increasing number of dry-eye patients in their daily practice, they should be familiar with quick, reliable, and less invasive diagnostic tests to manage the disease successfully.

## Conclusions:

We observed prevalence of dry eye among computer users of our institution. It was found to be related with number of working hours. Patients' education, deliberate ergonomics of computer use, including screen height, chair position, blinking exercises, glare protection and artificial tear drops use in order to minimize the symptoms of dry eye syndrome and prevent serious complications.
